# Propriety assessment model for life cycle operational global warming potential of apartment buildings in Korea using energy efficiency and energy effective area data

**DOI:** 10.1038/s41598-023-29142-6

**Published:** 2023-02-10

**Authors:** Hyunsik Kim, Hyojin Lim, Jeonghwan Kim, Seungjun Roh

**Affiliations:** 1 Department of Civil Engineering, Korea National University of Transportation, Chungju, 27469 Republic of Korea; 2 Department of Architectural Engineering, Hanyang University, Ansan, 15588 Republic of Korea; 3 School of Architecture, Kumoh National Institute of Technology, Gumi, 39177 Republic of Korea

**Keywords:** Energy science and technology, Engineering

## Abstract

In response to global warming, researchers worldwide are actively investigating various techniques and institutional frameworks to reduce the emission of greenhouse gases. Despite numerous life cycle assessment (LCA) studies indicating that global warming effects due to lifetime energy consumption are the greatest in the building operation stage, the absence of a standard global warming potential (GWP) report based on building energy usage makes it difficult to examine realistic GWP reduction directions. In South Korea, energy data for numerous buildings were collected through the Building Energy Efficiency Certification (BEEC) for several years, with data from apartment buildings receiving the most attention. GWP emissions were evaluated using the data through Green Standard for Energy and Environmental Design LCA. Here, we developed a model for apartment buildings to assess mutual propriety for GWP emissions (E) and energy effective area ratio (R_E_) during building operation to support the reduction of GWP emissions caused by lifetime operational energy consumption resulting from planning and design. We collected apartment BEEC data and used them to calculate the energy effective area ratio and GWP emissions of each building, which were then classified by energy use and source. Linear regression analysis was performed between R_E_ and E for each classification, and the derived regression equation was developed as a GWP assessment model for apartments. The applicability of the proposed model was examined through a case study, which confirmed that the model can be used to determine design directions for reducing GWP emissions for every energy in apartments.

## Introduction

In response to exacerbating global warming, researchers worldwide are actively investigating various techniques and institutional frameworks to reduce the emission of greenhouse gases. In the construction sector, authorities have established green building certification systems such as LEED in the United States, BREEAM in the United Kingdom, and Green Mark in Singapore, and are working to reduce the environmental impact and greenhouse gas (GHG) emissions produced by buildings^1^. In South Korea, the Green Standard for Energy and Environmental Design (G-SEED) was implemented in 2002, and following the global trend, authorities are working to reduce the environmental impact of the construction industry. Furthermore, life cycle assessment (LCA) was adopted as an additional scoring factor in G-SEED in 2016 to strengthen the system^2,3^. Based on the ISO-14040 series, building LCA categorizes the life cycle stages of a building into building material production, construction, operation, and dismantlement/disposal stage to evaluate the environmental impact of each stage and the total life cycle environmental impact. The assessment results of global warming potential (GWP), an environmental impact category, are a vital indicator for analyzing how the construction sector impacts global warming^3–5^. Several studies have reported that the GWP from energy use is the highest in the building operation stage among all life cycle stages, and it is necessary to identify energy-use-associated issues during the planning and design stages^6–10^.

GWP emissions in the building operation stage refer to the global warming load generated by energy types such as electricity and gas used to operate a building during its lifetime service and are calculated by applying the GWP energy intensity factor of each fuel to the energy consumption. As the GWP energy intensity factor varies greatly with the type of energy used, the energy type is critical for calculating GWP emissions. However, due to factors, such as structural characteristics of buildings and their economic feasibility, it is difficult to change the energy source to reduce the global warming load without any hindrance. For this reason, a realistic direction for reducing GWP must be presented when designing a building to supply necessary assessment standards for global warming loads based on previously calculated energy consumption and GWP emission data in the building operation stage.

Energy efficiency data is a major metric for a building’s energy consumption. In South Korea, the Building Energy Efficiency Certification (BEEC) system for apartments was initiated in 2001 and is currently being implemented for all building types. As of 2022, about 24,000 buildings have received energy certifications through this system^11,12^. BEEC divides the energy use of buildings into air cooling, heating, hot water supply, lighting, and ventilation, and it calculates the primary energy consumption per unit energy effective area, then assigns one of 10 grades ranging from 7 to 1+++^12^. The energy effective area calculated while estimating BEEC refers to the floor area in which the building’s air cooling, heating, hot water supply, lighting, and ventilation have an effect. Passive design can be performed by increasing the ratio of energy effective area within the building’s total floor area to thereby reduce energy load per area^13–15^.

With the recent spread of G-SEED nationwide, GWP assessment data of buildings is gradually accumulating^16^. Assuming a 50-year building life span, G-SEED LCA—whose data is representative of GWP emissions of buildings—calculates GWP from the building operation stage over the lifetime of a building, using the primary energy data of BEEC as basic data^17^. Currently, G-SEED LCA and BEEC data are very useful for presenting global warming impact standards for buildings at the national level. These standards can be used to improve the active design of buildings by increasing the efficiency of facilities that use energy or reducing the amount of directly consumed primary energy by using new and renewable energy^18–20^. However, previous studies are mostly limited to calculating the life cycle GWP of buildings and do not present standards to assess its propriety^21–24^.

**Figure 1:**
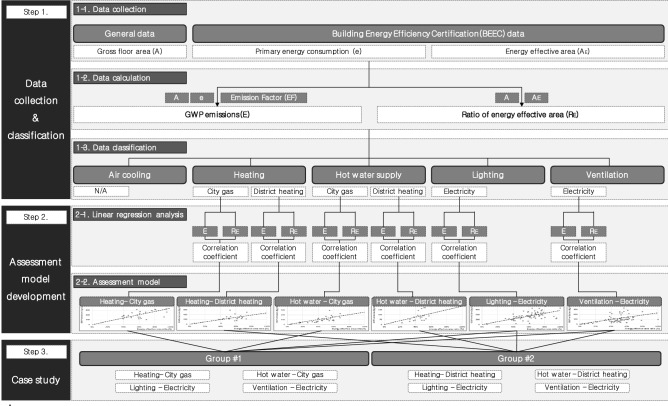
Research framework.

Focusing on apartment buildings—on which BEEC is most actively performed in South Korea—in this study, we developed a model using the G-SEED LCA method that calculates GWP from BEEC data to assess the mutual propriety of GWP emissions and energy effective area in the building operation stage with the aim of providing a method to help building designers to check the appropriateness of GWP emissions and energy effective area mutually during the planning and designing stage of apartments in Korea. First, to develop the model, this study collected BEEC data for apartments and used it to calculate the energy effective area ratio (R_E_) and GWP emissions (*E*) of each apartment. We then classified the calculated data by energy use and energy source, conducted a linear regression analysis between R_E_ and *E* for each classification, presented the derived regression equation as a GWP propriety assessment model for apartments, and examined the utility of the proposed model using a case study. Figure 1 presents the framework of this study.

## Materials and methods

### Data collection and classification

Data for this study were collected from 65 new apartment buildings that underwent BEEC evaluation from 2018 to 2021. The types of collected data consisted of gross floor and energy effective areas, as well as the primary energy consumption of each building. The gross floor area signifies the total floor area of each floor of a building and the energy effective area signifies the floor area where the active air cooling, heating, hot water supply, lighting, and ventilation effects reach within the total floor area. The energy effective area ratio of energy effective area to gross floor area was calculated using Eq. 1 based on the data of each building.

1 $$ R_E = \frac{A_E}{A}$$

where R_E_ is the energy effective area ratio (%), *A*_E_ is the energy effective area (m^2^), and *A* is the gross floor area (m^2^). In addition, GWP emissions according to the primary energy consumption of each building were derived based on the G-SEED LCA calculation method for environmental impact in the building operation stage^17^. Since the primary energy consumption data of BEEC is essentially a value representing the primary energy of the unit energy effective area, it must be converted into the unit floor area utilized when representing the environmental impact of a building. Accordingly, this study derived the GWP emissions through Eq. 2.

2 $$\it E_0 = \frac{e \times A_E}{A} \times {\text{EF}}$$

where *E*_*0*_ indicates the GWP emissions (kg-CO_2_eq/m^2^ year) per unit floor area per year of the building, *EF* is the emission factor for each energy source (kg-CO_2_eq/kWh), and *e* is the primary energy consumption (kWh/m^2^ year) collected in the BEEC. When applying *EF*s in G-SEED LCA, the IPCC1996 emission factors were used for the direct emission factors in our study. For indirect emission factors, the Korean National Life Cycle Inventory Database (LCI DB) or an LCI Database developed in another country was used. Since city gas is directly combusted within the building, both indirect and direct emissions were considered for its calculation, whereas only indirect emissions were considered for electricity and district heating. Since G-SEED LCA assumes that the lifespan of a building is 50 years, this value was applied to calculate *E*, which indicates the GWP emissions (kg-CO_2_eq/m^2^) based on the lifetime operation of a building, as shown in Eq. 3.

3 $$ {\text{E}} = {\text{E}}_0 \times 50$$

Data classification was then performed based on energy use and energy source to analyze the relationships between R_E_ and* E* calculated for 65 apartments. BEEC in South Korea classifies the energy use of buildings into air cooling, heating, hot water supply, lighting, and ventilation—according to the standards of BEEC and zero energy building certification^25^. However, unlike in other building types, air cooling facilities in apartments are not installed all at once during the building construction stage but are installed and operated individually for each household after construction. As such, BEEC does not assess energy consumption due to air cooling in apartments. Moreover, among the energy sources for buildings—city gas, district heating, and electricity—city gas or district heating are the primary energy sources for heating and hot water supply in apartments, whereas electricity is used for lighting and ventilation. Therefore, we classified the data as shown in Table 1 and performed an analysis.

**Table 1:** Classification of BEEC assessment data by energy use and energy source.

Classification system		Number of data samples (number of cases)
Energy use	Energy source	
Heating	City gas	47
	District heating	18
	(Total)	65
Hot water supply	City gas	47
	District heating	18
	(Total)	65
Lighting	Electricity	65
Ventilation	Electricity	65

### GWP assessment model development

This study used the classified data to analyze the linear correlation between *E* and R_E_ for each energy use and source, and then presented the highly correlated linear regression equations as GWP assessment models for apartments. Since *E* has a value of 0 when R_E_ is 0, regression equations passing through the origin were easily derived. To ensure the reliability of the regression equations during this process, the regression analysis was performed within the 95% confidence range of the data.

**Figure 2:**
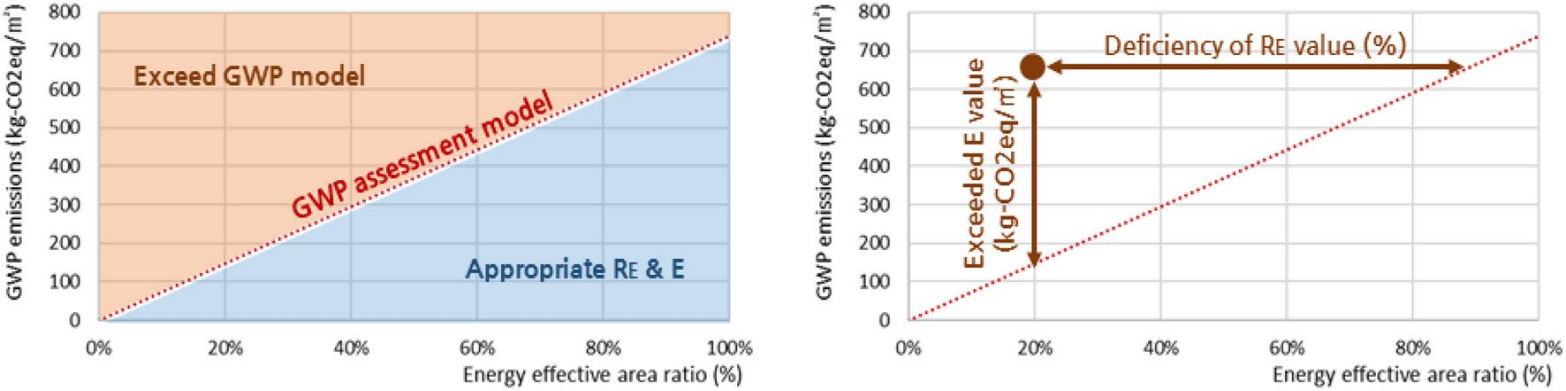
Use of global warming potential (GWP) assessment model.

The final derived GWP assessment model calculated the appropriate GWP emissions according to the energy effective area ratio based on the energy use and energy source of each apartment. Using the equation of the GWP assessment model, the model was then visualized to assess the propriety between R_E_ and *E* of each apartment, which was then used in a further assessment, as shown in Fig. 2. Additionally, the average R_E_ for each energy use and source was calculated and presented in each model to support the decision of whether the energy effective area is sufficient compared to the gross floor area or not.

### Case study

Based on the GWP assessment model, a case study was performed to assess the mutual propriety between R_E_ and *E* based on the energy use of each building. The targets in the case study were two groups of two apartments: Group #1 used city gas for heating and hot water supply, and Group #2 used district heating for the same purposes. Real BEEC assessment information for each building was used to conduct the assessment. Finally, the propriety between R_E_ and *E* was analyzed, and the applicability of the GWP assessment model was examined.

## Results

### Classified data

**Figure 3:**
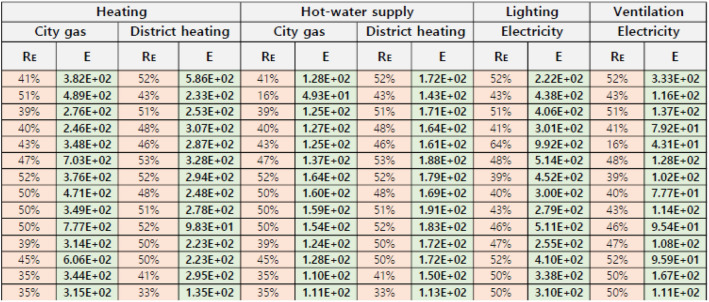
Classified dataset (partial).

Using the collected BEEC data, this study classified the calculated R_E_ and *E* values per unit floor area based on energy usage and source for 65 apartments, which formed the dataset shown in Fig. 3. Using the classification system of the dataset, a regression analysis of R_E_ and *E* by energy use and energy source was performed.

### GWP assessment model

**Figure 4:**
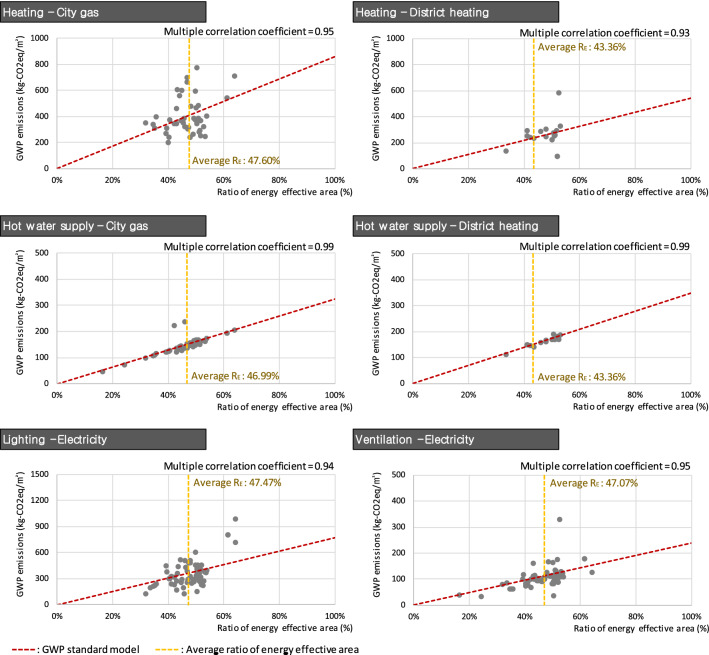
Linear regression analysis results by energy use and energy source of the apartments.

Table 2 shows the results of deriving the GWP assessment model through a linear regression analysis classified by energy use and energy source of the buildings. In the process of deriving the model, the multiple correlation coefficients between R_E_ and *E* and the model equations for every energy use and energy source exceeded 0.94, indicating high linear correlations. Particularly high correlation coefficients of 0.99 were obtained for the use of both district heating and city gas for hot water supply. This demonstrates that each model equation may be used to confirm the mutual propriety between R_E_ and *E* of the apartments. Figure 4 presents a visualization of the correlation analysis results and model.

**Table 2:** GWP assessment standard model equation by energy use and energy source of the apartments.

	City gas	District heating	Electricity
Heating	y = 860.01x	y = 541.24x	N/A
Hot water supply	y = 324.78x	y = 348.11x	N/A
Lighting	N/A	N/A	y = 775.58x
Ventilation	N/A	N/A	y = 239.10x

*N/A* not available, *x* energy effective area ratio (%), *y* GWP emissions per gross floor area (kg-CO_2_eq/m^2^).

### Reliability examination of GWP assessment model

The statistical performance of each GWP assessment model equations were analyzed as shown in Table 3 to check the reliability of each model. Among the statistical indexes calculated in this study, mean absolute percentage error (MAPE) shows the prediction accuracy of each model equation most efficiently. As a result, low MAPE of 5.37% and 2.93% were obtained respectively for the use of city gas and district heating for hot water supply. Moreover, the MAPE of the ventilation model equation also reached a reasonable level of 19.61%. However, the heating and lighting model equations all exceeded 20% of MAPE. High MAPE could be occurred by the difference in the energy efficiency of the energy system installed. Therefore, the efficiency of heating and lighting systems used for each sample in this study should be identified and classified in more detail in further studies.

**Table 3:** Statistical performance of GWP assessment standard model.

	City gas (RMSE/MAE/MAPE)	District heating (RMSE/MAE/MAPE)	Electricity (RMSE/MAE/MAPE)
Heating	131.58/103.42/26.81	99.40/62.76/34.05	N/A
Hot water supply	19.44/8.90/5.37	6.08/4.80/2.93	N/A
Lighting	N/A	N/A	133.70/100.09/31.77
Ventilation	N/A	N/A	34.94/20.27/19.61

*N/A* not available, *RMSE* root mean squared error, *MAE* mean absolute error, *MAPE* mean absolute percentage error (%).

### Case study results

To conduct the case study, the energy sources for each energy use of the apartments were divided into two groups. Group #1 consisted of apartments that use city gas as the energy source for heating and hot water supply, and Group #2 consisted of apartments that use district heating for the same purposes. Both groups used electricity for lighting and ventilation. Two target apartments from each group were selected and analyzed. Table 4 presents the BEEC information of each apartment collected for the case study.

**Table 4:** Energy use information of target apartments for case study.

Case study of group #1	Case 1–1	Case 1–2
Floor area (m^2^)	39,907.73	97,621.25
Energy effective area (m^2^)	23,795.89	48,813.31
Primary energy consumption (kWh/m^2^ year)		
Heating	47.9	51.8
Hot water supply	25.8	26.3
Lighting	26.5	24.7
Ventilation	10.9	8.4

Case study of group #2	Case 2–1	Case 2–2
Floor area (m^2^)	42,817.92	148,223.76
Energy effective area (m^2^)	21,959.90	75,794.22
Primary energy consumption (kWh/m^2^ year)		
Heating	20.3	38.4
Hot water supply	26.9	26.4
Lighting	44.3	27.4
Ventilation	10.0	9.1

1. Group #1: Heating (city gas), Hot water supply (city gas), Lighting and Ventilation (electricity).

2. Group #2: Heating (district heating), Hot water supply (district heating), Lighting and Ventilation (electricity).

**Figure 5:**
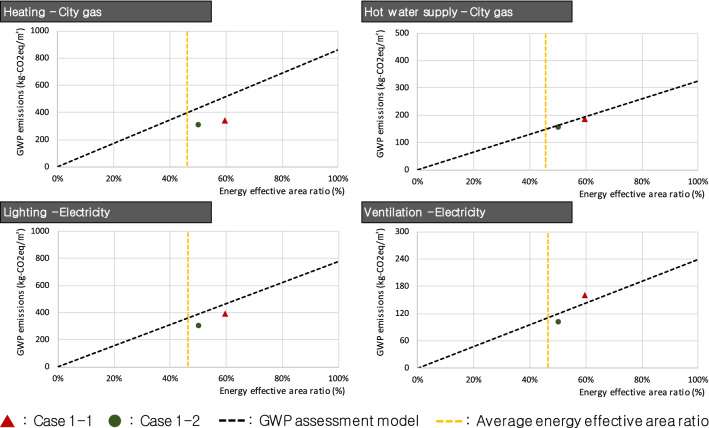
Case study results of Group #1.

Figure 5 shows graphs of R_E_ and *E* per unit floor area by energy use for Group #1. Both apartments in Group #1 showed high R_E_ level exceeding the average and generally appropriate *E* for heating, hot water supply, and lighting; however, the *E* in Case 1–1 had a somewhat higher trend compared to the GWP assessment model for ventilation. Case 1–1 has an R_E_ of 29.63%, thus the appropriate GWP emissions should have been 142.57 kg-CO_2_eq/m^2^. However, the calculated *E* were higher than the appropriate value, at 160.89 kg-CO2eq/m^2^ (12.85% higher than the appropriate GWP emissions). To address this limitation, R_E_ should be increased by approximately 7.66% by expanding the energy effective area or *E* reduced by about 18.32 kg-CO_2_eq/m^2^ by improving the ventilation energy efficiency. In this case, reducing *E* could be a more proper way to solve the problem than increasing R_E_ because the value of R_E_ is already high enough to exceed the average.

**Figure 6:**
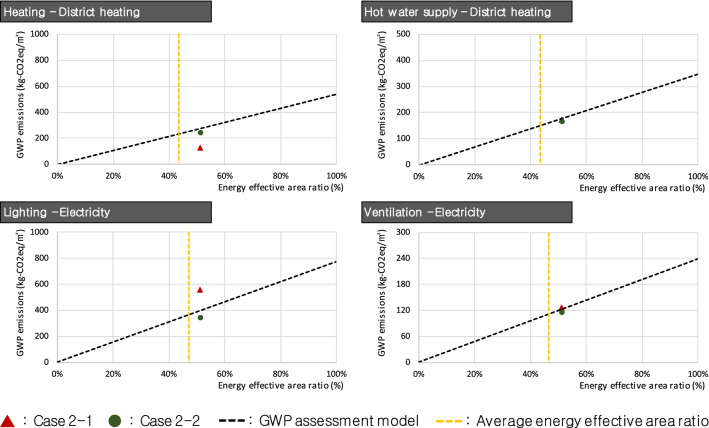
Case study results of Group #2.

Next, graphs of R_E_ and *E* by energy use for Group #2 were derived, which are presented in Fig. 6. Unlike Group #1, Group #2 showed generally appropriate *E* for heating, hot water supply, and ventilation; however, Case 2–1 showed a somewhat high *E* compared to the GWP assessment model for lighting. Case 2–1 had an R_E_ of 51.29%, thus the appropriate GWP emissions should have been 436.54 kg-CO_2_eq/m^2^. The calculated *E*, however, were 125.91 kg-CO_2_eq/m^2^ higher than the appropriate value, at 562.44 kg-CO_2_eq/m^2^. Moreover, the appropriate R_E_ value for the calculated *E* was 66.01%, whereas the actual R_E_ value was about 14.79% lower. This limitation could be addressed by improving the energy efficiency of lighting facilities by the use of electricity or through a redesign to improve the energy effective area ratio. In this case, reducing *E* could be a better way to solve the problem because the value of R_E_ is already high enough to exceed the average.

Through the case study of Groups #1 and #2 to examine the applicability of the GWP assessment model, this study confirmed that it is possible to easily assess the propriety of GWP emissions according to energy effective area by energy use in an apartment. It also demonstrated that design directions for environmental improvement could be determined using the assessment results.

## Discussion

This study developed and proposed a GWP assessment model to assess the propriety of GWP emissions based on the energy effective area ratio due to energy use in the operation stage, which is the life cycle stage of apartments with the highest GWP. The methodology used in this study was reliable in that the model was derived by statistically utilizing building energy data officially assessed through the BEEC system of South Korea, and it is meaningful in that the energy consumption propriety of an apartment in the context of global warming can only be confirmed using BEEC data. The model and assessment methodology developed in this study are expected to mainly serve as tools to support decision-making when devising plans for energy-using facilities, spaces, and new apartments in the future. Furthermore, through further studies, the model development method in this study can be used to propose assessment models for not only apartments but also buildings of relatively standard uses, such as commercial buildings and schools. However, the number of data samples used to derive the model via regression analysis in this study must be expanded, and models which show high error rate should be improved by considering the actual energy efficiency of the energy system installed in future studies to improve reliability. Moreover, major factors considered in previous building energy studies such as thermal performance of building envelope and transmittance losses, HVAC system with heat recovery potential, and infiltration rate should be considered along with the energy effective area in further studies to develop an actual solution of reducing GWP caused by energy consumption. With BEEC being actively conducted on new apartments in South Korea, further researches will be conducted to improve the assessment model.

## Conclusions

In this study, we developed a model for apartment buildings to assess the mutual propriety of GWP emissions and energy effective area ratio in the building operation stage, with the purpose of reducing GWP emissions originating from lifetime operational energy consumption derived from building planning and design. The following conclusions were drawn:

1. BEEC data for 65 apartments were collected and used to calculate energy effective area ratio (R_E_) and GWP emissions (*E*) per unit floor area for each apartment. The calculated data were then classified by energy use (heating, hot water supply, lighting, ventilation) and energy source (city gas, district heating, electricity) of the apartments and used to construct a data set for regression analysis.
2. Using the classified data set, linear regression equations passing through the origin for *E* and R_E_ values were derived for each energy use and energy source, and the regression performance were analyzed. Highly correlated linear regression equations were then proposed as GWP assessment models for apartments. However, the heating and lighting model equations showed mean absolute percentage error exceeding 20%.
3. To examine the applicability of the proposed model, we randomly selected five apartments that used city gas as the energy source for heating and hot water supply, and five apartments that used district heating as the energy source for the same. Then, based on the GWP assessment model by energy use and energy source of each apartment, a case study was conducted to assess the mutual propriety between the energy effective area and GWP of the apartments.
4. Finally, this study confirmed that the propriety of *E* and R_E_ values for each energy use in an apartment can be examined using the proposed GWP assessment model, through which design directions to reduce GWP emissions can be determined.

The study’s findings are expected to mainly serve as tools to support decision-making when devising plans for energy-using facilities, spaces, and new apartments in the future, as well as basic data to build assessment models for not only apartments but also buildings of other uses such as commercial buildings and schools.

## Data Availability

All data generated or analyzed during this study are included in this published article.
